# Central and Peripheral Attention in Virtual Reality: Test of Visual Efficiency for Concussion Detection

**DOI:** 10.1089/jmxr.2023.0001

**Published:** 2024-01-25

**Authors:** Jennifer C. Reneker, William A. Pruett, W. Cody Pannell, Michael Brown, Ryan M. Babl, Heather L. Shirley, Yunxi Zhang

**Affiliations:** 1 Department of Population Health Science, University of Mississippi Medical Center, Jackson, Mississippi, USA.; 2 Department of Physiology, Biophysics, and University of Mississippi Medical Center, Jackson, Mississippi, USA.; 3 Department of Physical Therapy, University of Mississippi Medical Center, Jackson, Mississippi, USA.; 4 School of Applied Sciences, The University of Mississippi, Oxford, Mississippi, USA.; 5 Department of Data Science, University of Mississippi Medical Center, Jackson, Mississippi, USA.

**Keywords:** virtual reality, peripheral vision, concussion

## Abstract

**Introduction:**

In complex and fast-paced environments, central and peripheral vision are imperative to respond to changes in the environment and ensure personal safety. Measures of vision have become key interests in research exploring biomarkers of concussion. The purpose of this project was to describe differences in performance between individuals with and without an acute concussion on a novel test of peripheral vision delivered through a virtual reality head-mounted device (HMD).

**Methods:**

This was a matched case–control study, including 13 participants with and 26 participants without a concussion. The peripheral vision test required visual focus on a center object and use of peripheral vision to identify and select the matching side object with the hand controller. After each selection (i.e., trial), the shapes and colors of all the objects changed and the side objects moved further into the periphery. This was done for 60 trials. Test performance was compared between matched pairs on the following outcomes: number of trials where the eyes remained on the central object (i.e., a valid trial), correctness among valid trials, and mean response time for selection. Effect sizes were used to describe the magnitude of difference between group means.

**Results:**

Moderate effects were found, indicating a greater number of valid trials, for the test overall (*d* = 0.46; 95% CI 0.04–0.80), with small-to-moderate effects in each subsection by the participants without a concussion than those with a concussion. Among the valid trials, there was no difference in correctness of peripheral selection between groups. Differences in response times between groups became larger as the test progressed; those with a concussion were significantly slower that than those without a concussion in the second half of the tests (adjusted *p*-values = 0.012–0.024). Large effects were observed across all measures of response time, with the largest effects observed in the trials at the furthest periphery (trials 46–60 *d* = 1.75; 95% CI 0.98–2.52).

**Conclusion:**

This novel test in a virtual reality HMD was able to corroborate findings with previously published studies on this topic. Our results elucidate deficits in functional peripheral vision processing after concussion. This is important when designing rehabilitative programs to target specific deficits.

## Introduction

The human visual field is made up of two parts, central and peripheral. Relatively speaking, if the total visual field is 170°, roughly 70° is considered central vision and 100° is peripheral vision.^1,2^ Each eye's visual field can be further broken down into central vision (0–18° of the visual field); near-peripheral, which is closest to your central vision (18–30° of the visual field); midperipheral (30–60° of the visual field); and far-peripheral, which is the farthest laterally (60–110° of the visual field).^3^

The central and peripheral components of vision are quite different in their cellular composition,^4^ role in visual processing,^5,6^ and neural processes contributing to visuomotor function.^7–9^ Although the visual cortex processes central and peripheral information simultaneously, the information is processed independently, enabling the two parts of vision to be used and interpreted differently.

Peripheral vision allows us to see and respond to objects lateral to our body,^10^ gives us a sense of spatial orientation,^9^ assists in postural control and balance,^11^ provides information about the scene to get the gist of the environment,^3^ and enables us to view objects moving toward or around us.^10^ Humans remain consciously aware of what is being processed through their central vision, while they are subconsciously aware of what is being processed through their peripheral vision until there is a reason to direct conscious attention to it.^12,13^ In other words, the cortical processing of peripheral information causes a shift in central focus and attention, when needed.^14,15^

In complex, changing, dangerous, or fast-paced environments, both fields of vision are imperative to respond to changes in the environment, plan goal-directed movement, avoid potential impacts, and ensure personal safety.^16^ Given these roles of peripheral vision, it is apparent that peripheral vision is exceedingly important for participation in contact sports, while operating motorized vehicles or aircraft, and during military operations.

Measures of vision and oculomotor control have become key interests in research exploring biomarkers of mild traumatic brain injury/concussion.^17–23^ Most eye-tracking research is focused on measures requiring central vision.^18,21,24^ Fewer studies have been conducted where use of peripheral vision was required.^25,26^ The purpose of this project was to describe differences in performance between individuals with an acute concussion and controls on a novel test of peripheral vision delivered through a virtual reality (VR) head-mounted device (HMD) and to discuss the potential value of this test in the detection of concussion.

## Methods

### Study design

This was a matched case–control study, nested within a large cohort. Enrollment and data collection for each participant occurred on one day with no follow-up between October 2020 and February 2022. This project was approved and overseen by the University of Mississippi Medical Center Institutional Review Board. A reliance agreement was used to provide research oversight with the partnering institutions. This research report was written according to The Strengthening the Reporting of Observational Studies in Epidemiology (STROBE) guidelines, with the extension for health care simulation research.^27^

### Settings

Participants aged 18–40 years with and without an acute concussion were enrolled from four universities in Mississippi and one in Louisiana (The University of Mississippi Medical Center, The University of Mississippi, Mississippi State University, Mississippi College, and Louisiana State University). This included the general student body and the scholarship athletes.

### Participant eligibility

Through convenience sampling, participants were enrolled into one of two groups, those with and without an acute concussion. For the control participants (i.e., those without an acute concussion), researchers recruited participants in common areas on each campus (campus recreation, student centers, etc.). Multiple dates of recruitment occurred at each campus over the data collection period.

The determination of individuals with acute concussion was based on physician diagnosis. Potential participants with an acute concussion were invited to participate in the study at one of the participating universities after receiving a diagnosis of a concussion. Individuals with an acute concussion were considered eligible as long as they had a physician diagnosis (regardless of diagnostic approach) and were not released for return to play/return to typical activity by their physician at the time of enrollment.

Regardless of group, all potential participants were between 18 and 40 years of age who (1) were able to stand and maintain balance while completing the tests, (2) consumed food and/or beverage within 12 hours before test completion, and (3) slept >1 hour within the 24 hours before test completion. Individuals with a seizure disorder, photosensitivity, or any other persisting disease or condition, which would impact their ability to stand safely while wearing a HMD and persons who were unable to understand the verbal instructions for test completion because of a cognitive deficit, were excluded.

If an investigator had reason to believe that a participant was not safe to complete the tests or if the participant's symptom responses, either at the beginning of the tests or throughout completion of the tests, raised concerns, the individual was excluded or withdrawn.

### Variables and data sources

After enrollment, participant data on self-reported demographic (i.e., gender, age, race, and ethnicity), medical (e.g., history of previous central nervous system injuries, and history of COVID-19 infection), and personal/social history (e.g., number of hours of sleep, handedness, participation in sports, education, and occupation) were collected through a REDCap survey on an iPad. REDCap is an encrypted cloud-based software for storage and management of research data.

Sensors in the HMD and hand controller, including an eye tracker for both eyes, accelerometers, and gyroscopes, registered measurements of eye movement, positional movement, and selection of objects in the HMD environment. For this test, the sensor-based measurements were recorded at 90 Hz and included left and right eye positions, rotation, and openness; and HMD position and focus position (all the aforementioned were measured in *x*, *y*, and *z* axes). The identification of the focus object, corresponding peripheral object position for each trial, and the object selected in each trial were recorded. A timestamp for when the focus object was first visible and when the user selected with the hand controller was used to calculate the response time for selection.

### Participant orientation

Each participant was individually oriented to the HMD, the physical environment, and the manual controller (one hand only based on their preference). They were assisted into the headset and the application was started by the examiner. The headset was adjusted to ensure that the central visual field was optimally clear to the user (i.e., the sweet spot). The headset was not calibrated for each user. In addition, eyeglasses or corrective contacts worn by the participant at the time of enrollment were encouraged to be worn in the headset during testing.

### Simulator type

The HTC Vive Pro Eye HMD provided the virtual environment and was tethered to an Alienware, Area 51 laptop computer, which ran the software application and collected the sensor-based data. The software application, called the Virtual Immersive Sensorimotor Test of Neurological Impairment Detection (VIST Neuro-ID), was created by members of the research team for this project. The test of peripheral vision was the fourth in a test set of eight discrete tests, measuring different sensorimotor proficiencies. The software also included an examiner dashboard, which was used to control the HMD environment, select the tests, observe the participant's performance during the tests, and track symptoms over time.

### Simulation environment

Research activities occurred in common areas on each campus of the participating universities, which were easily accessible by potential participants and provided minimal distraction to participants while in the headset. The VR system was set up as “standing only,” meaning that there was a single location of stance for the tests, which was prespecified for all participants by the study team. Before participant enrollment, the physical testing environment was cleared of any obstacles to ensure participant safety.

### Virtual test environment

The software was designed to run as a self-contained testing environment, operated by a human examiner. The examiner could pause the software and provide additional instructions if a participant did not understand the instructions or could stop a test for safety reasons. In addition, if a participant recorded increased symptoms in-between tests, the examiner verbally asked whether the participant agreed to continue. The examiner logged notes of any additional instructions or interactions that the participant required. Otherwise, the examiner did not interact with the participant during the tests or provide any feedback to the participant during or after a test.

The virtual environment included a static gray “room” consisting of a 3D grid extending into the distance, providing a top and bottom. The VR environment was free of visual and audio distractions, except for those required for test completion.

### Virtual test scenario

**FIG. 1.**
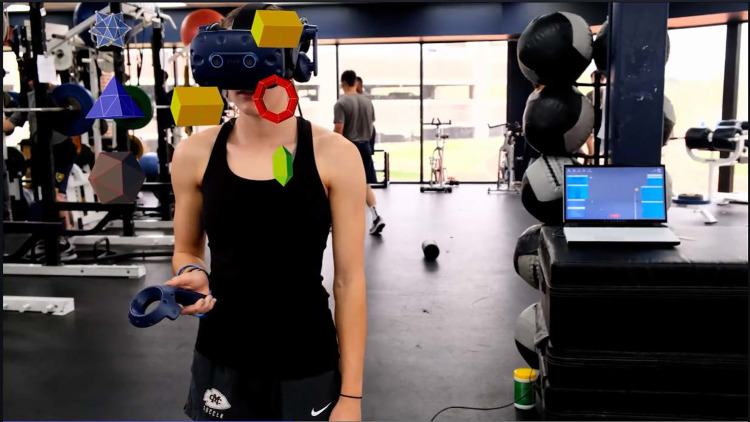
Animated representation of the virtual environment representing the display in the headset.

**FIG. 2.**
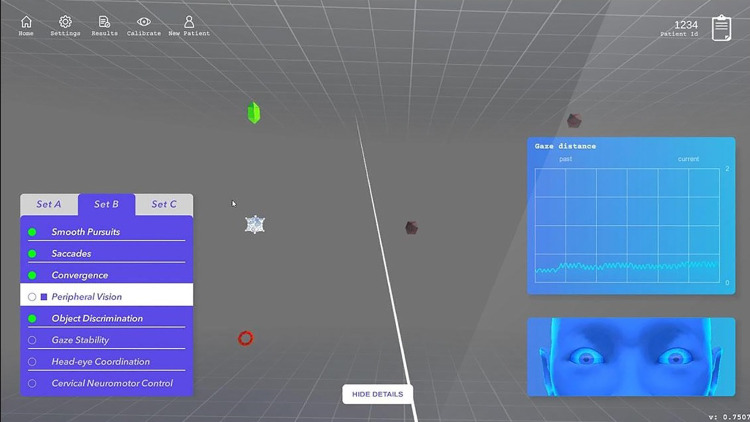
Display of the peripheral vision test as displayed on the examiner dashboard.

The peripheral vision test included one central focus object and six peripheral objects, three each on the left and right sides in a vertical column (up, center, and down). The test required maintenance of focus on the center object and use of peripheral vision to identify and select the matching side object with the hand controller (Figs. 1 and 2). After every selection, the shapes and colors of all the objects changed and the side objects moved further into the periphery. If a selection was not made within 2 seconds, the objects changed automatically. This was done for 60 repetitions (i.e., trials).

In addition, there was a 0.15 second timeout at the beginning of a new condition so that the participant was not able to input a selection. This feature was implemented to prevent accidental failure of two conditions in a row by attempting to input a selection for the prior trial just after transitioning to the next trial.

The peripheral objects began at an apparent distance of 1.0 m in front of the participant in a Unity world coordinate system, and 10° from the fixed center of vision. Thus, the test began with the lateral objects in the field of central vision. With each selection, the peripheral objects advanced 0.75° radially from the fixed center of vision. Once they reached the maximum of 40° from midline in both horizontal and vertical deviations, they stopped moving radially. Thus, the test concluded with the lateral objects in the midperipheral field of vision. Once at this location, the central and peripheral shapes continued to change at random with each trial until 60 trials were completed.

Before the test, the participant was provided with animated instructions for test completion. The audio instruction for peripheral vision states, “You will see a central object and a series of objects on the sides. Keep your eyes on the center object, use your side-vision (or peripheral vision) to identify the same object on the side, and select the matching object with the hand controller as quickly you can. Remember, keep your eyes on the center object.” An animated avatar and objects are displayed during these verbal instructions. This was followed by a “ready” activation button, which set the *x*, *y*, and *z* axes for each test, and then an audiovisual 3-2-1 countdown.

### Statistical methods

A propensity score matching was performed to identify nonconcussed participants who had similar baseline covariates to concussed participants. First, the propensity score was estimated with a multivariate logistic regression model. Gender, race, number of hours slept the night before the test, and varsity athlete status were considered predictors. Optimal 1:2 matching between concussed and nonconcussed participants was implemented based on the logit of propensity scores, with an absolute distance <0.5.

After matching, all predictors were assessed between the groups using a *t*-test for normally distributed continuous variables and Fisher's chi-squared test for categorical variables with expected counts <5. With balanced predictors between groups, we identified the two comparable groups for the analysis on outcome measures.

We compared the test performance between matched pairs on the following outcomes: number of trials where the eyes remained on the central object (i.e., a valid trial), correctness among valid trials (i.e., based on the central object presented, how often the correct peripheral object was selected with the hand controller), mean response time without penalty for eye movement off the center (i.e., cheating), and mean response time with a penalty for eye movement off the center. The penalty assigned was the maximum allowable time between trials. For each outcome, the overall performance was evaluated across trials 2–60 as well as quartile sections of trials 2–15, 16–30, 31–45, and 46–60.

As we have multiple nonconcussed participants for each concussed participant, comparisons were conducted between average test results of matched nonconcussed participants and test results of concussed participants through paired *t*-test or Wilcoxon signed-rank test depending on the normality of the variable distribution. A Holm–Bonferroni *ad hoc p*-value adjustment was applied to subsection tests (trials 2–15, 16–30, 31–45, and 46–60) of each outcome. Effect sizes were calculated, following Cohen's *d* for paired *t*-tests and Rosenthal for Wilcoxon signed rank tests, to describe the magnitude of the differences between groups.^28^

## Results

In total, 608 participants were included in the cohort. Within this, 24.4% are non-White, 49.7% are female, and 25.8% are varsity collegiate athletes. Of the 608 total participants, all of those with an acute concussion who completed the peripheral vision test were included in this analysis. Therefore, within the matched sample, 13 participants with a concussion and 26 participants without a concussion were included in the analysis (Table 1). Of these, 38.5% were Black, 36% were female, and 84.6% were varsity collegiate athletes. After matching, each concussed participant was paired with two nonconcussed participants and showed balance characteristics between matching pairs.

**Table 1.** Participant Baseline Characteristics

	Total nonconcussed sample (*n* = 594)	*Matched nonconcussed (* *n* * = 26)*	*Acute concussion (* *n* * = 13)*	*Matched sample weighted * *p* *-value*
Race, no. (%)				
Black or African American	78 (13.1)	10 (38.5)	5 (38.5)	1.000^a^
White	449 (75.6)	16 (61.5)	8 (61.5)	
Gender, female, no. (%)	67 (11.3)	10 (38.5)	4 (30.8)	0.680^a^
No. of hours slept in the past 24 hours, mean (SD)	7.1 (1.3)	6.6 (1.6)	6.9 (1.4)	0.633^b^
Varsity athlete	295 (49.7)	22 (84.6)	11 (84.6)	1.000^a^

a Fisher's exact test.

b *t*-Test.

For performance on the peripheral vision test, significant differences were not observed in the number of valid trials; however, moderate effects were calculated for the test overall [58.23 (0.67) vs. 53.85 (8.19); *d* = 0.46 (0.04, 0.80)] and in the trials in the furthest periphery (trials 46–60; mean 14.81 vs. 12.77; *d* = 0.54 95% CI (0.07–0.83), consistently in favor of those without a concussion (Table 2). Among the valid trials, there was no difference in correctness of peripheral selection between groups overall or for any of the subsections.

**Table 2.** Matched Analysis

		*Nonconcussed (* *n* * = 26)*	*Acute concussion (* *n* * = 13)*	*Raw * *p* *-value*	*Holm–Bonferroni adjusted *p*-value*	Effect size (95%CI)
No. of valid trials	Overall	58.23 (0.67)	53.85 (8.19)	0.092^a^		**0.46 (0.04 to 0.80)**
	Trials 2–15	13.85 (0.32)	13.54 (0.97)	0.375^a^	0.750	**0.18 (0.02 to 0.62)**
	Trials 16–30	14.81 (0.33)	14.08 (2.50)	0.594^a^	0.750	**0.01 (0.01 to 0.67)**
	Trials 31–45	14.77 (0.33)	13.46 (2.26)	0.134^a^	0.402	**0.35 (0.02 to 0.76)**
	Trials 46–60	14.81 (0.25)	12.77 (3.59)	**0.020** ^a^	0.080	**0.54 (0.07 to 0.83)**
Correctness among valid trials	Overall	0.83 (0.08)	0.87 (0.14)	0.526^b^		−0.39 (−1.06 to 0.28)
	Trials 2–15	0.83 (0.12)	0.91 (0.15)	0.229^b^	0.916	−0.61 (−1.29 to 0.07)
	Trials 16–30	0.85 (0.09)	0.87 (0.13)	0.817^b^	1.000	−0.19 (−0.85 to 0.48)
	Trials 31–45	0.84 (0.10)	0.85 (0.18)	0.897^b^	1.000	−0.07 (−0.74 to 0.59)
	Trials 46–60	0.81 (0.11)	0.85 (0.17)	0.492^b^	1.000	−0.31 (−0.98 to 0.35)
Mean response time (with cheating permitted)	Overall	864.44 (84.59)	1039.83 (219.67)	**0.012** ^b^		**1.23 (0.51 to 1.94)**
	Trials 2–15	934.39 (139.29)	1048.28 (247.47)	0.094^b^	0.094	**0.63 (0.05 to 1.31)**
	Trials 16–30	833.26 (108.73)	1024.78 (237.75)	**0.018** ^b^	**0.036**	**1.18 (0.47 to 1.18)**
	Trials 31–45	846.91 (100.97)	1051.94 (236.85)	**0.006** ^b^	**0.024**	**1.29 (0.57 to 2.02)**
	Trials 46–60	847.84 (68.87)	1034.89 (192.16)	**0.007** ^b^	**0.024**	**1.52 (0.77 to 2.26)**
Mean response time with penalized cheating (2 seconds)	Overall	877.37 (86.39)	1107.33 (250.75)	**0.006** ^b^		**1.44 (0.70 to 2.18)**
	Trials 2–15	941.74 (137.65)	1073.93 (256.57)	0.078^b^	0.078	**0.72 (0.03 to 1.40)**
	Trials 16–30	846.47 (116.89)	1064.19 (293.42)	**0.029** ^b^	0.058	**1.13 (0.42 to 1.84)**
	Trials 31–45	862.66 (99.28)	1125.91 (266.1)	**0.004** ^b^	**0.012**	**1.53 (0.78 to 2.28)**
	Trials 46–60	862.89 (74.81)	1163.05 (281.71)	**0.003** ^b^	**0.012**	**1.75 (0.98 to 2.52)**

Bold *p*-values indicate significant differences between groups; bold confidence intervals of effect sizes indicate significant effects.

a Wilcoxon signed rank test.

b *t*-Test.

There was a significantly longer response time overall when cheating episodes were penalized [mean 864.44 ms (SD 84.59) vs. 1039.83 ms (SD 219.67)] and were not penalized [mean 877.37 ms (SD 86.39) vs. 1107.33 ms (SD 250.75)] for the nonconcussed and concussed groups, respectively. When cheating was and was not penalized, significant differences in mean response time between groups were detected in the subsections of trials 31–45 and 46–60, and the differences in response time between groups became larger for trials in the furthest periphery. Large differences in the mean response times between groups overall (*d* = 1.23–1.44) were observed, consistently favoring the nonconcussed group. These effects became larger with trials in the furthest periphery.

## Discussion

This single test of functional peripheral vision on a matched sample of concussed cases and nonconcussed controls demonstrated several notable results. First, moderate differences in the number of valid trials were observed in the overall test and in the last 25% of the test between those with and without a concussion, meaning that individuals with a concussion had to shift their gaze laterally to locate and/or select the object more frequently that controls. We hypothesize that this could potentially indicate a decreased ability to (1) perceive the location of peripheral objects, (2) use peripheral vision to direct the upper extremity to the target, or (3) efficiently process concurrent central and peripheral visual information, requiring the use of central vision to locate the correct object.

This may be understood in light of findings, which have shown that transmission of central and peripheral information through neural networks of white matter tracts for action–control in open skills is more efficient in elite athletes than in novice athletes.^29^ Because it is well known that a concussion disrupts long tracts, our result is not surprising. Our VR test is unpredictable, requires decision making with speed, and a perception–action couple, making it an open skill. It is, therefore, likely that the expected transmission efficiency is impaired because of the damage to the white matter tracts, explaining the gaze shift to complete the task in the furthest periphery of the test.

This also provides evidence supportive of a second finding, which is that individuals with a concussion required upward of 200 ms more time than the nonconcussed controls to select the matching object in the periphery for the lateral 50% or 75% of the test, with and without a cheating penalty, respectively. It should be noted that within the first 25% of the test, the objects on the left and right sides were initially within the field of central vision (i.e., the objects started 10° off center). After the first 25% of trials in the test, visuospatial attention processing, using central (i.e., endogenous attention allocation) and peripheral (i.e., exogenous attention allocation) visuospatial cues were required to complete the test.^30^ In support of the findings in this present study, deficits of both processing mechanisms have been described previously in individuals with a concussion compared with controls,^31^ contributing to increased central and peripheral vision reaction times,^25,26^ with peripheral vision reaction times being more impaired than central vision reaction times after a concussion.^25^

A final interesting finding is that within the valid trials (i.e., noncheating trials), our participants with a concussion did not make more selection errors than the nonconcussed individuals. Together with the prolonged response time, we believe this indicates that after a concussion, peripheral vision perception of color and shape was not deficient, but the cortical processing time is delayed.

It should be noted that this type of test is not a typical component of a concussion screening or examination in traditional settings, and to our knowledge, there is no routinely used clinical correlate to the scenario we developed in the VR environment. Functionally, this test uses techniques and components routinely implemented in traditional perimeter or visual field testing,^2^ including the use of different shapes and colors and the importance of the individual maintaining gaze on a central object.

Some key differences between this test and traditional techniques are that this test was not designed to map peripheral vision and there is no change in luminosity for the various objects. This test did not engage far-peripheral vision, as the peripheral objects advanced to a maximum of 40 radial degrees from midline. In addition, this test requires participants to use central vision to identify the central object based on its shape and color and then use their peripheral vision to locate that same object in the periphery.

When identified, an upper extremity motor component is used to select the peripheral object, all while maintaining fixed gaze on the central object. This adds several elements of complexity, whereby the neurological processes that are likely contributing to performance on the test include peripheral visual acuity and central–peripheral shifting,^26^ visuospatial abilities,^32,33^ visual attention,^22^ and neural processing speed.^34,35^

### Limitations

Use of a convenience sample comes with inherent risk of limited generalizability and sample bias. Within the analytic sample, a high majority of the participants were college athletes, making these findings most applicable to athletes. Furthermore, the participants did not complete any standard clinical assessment of field of vision, making it possible that some of the differences between these groups could have come from pure differences in absolute visual field. Because the peripheral objects only moved into the midperiphery field of vision, the sample included generally neurologically healthy college students, and were predominantly collegiate athletes, we believe that our participants most likely fell within the established normative range for human vision and this would not account for the results presented here.

An additional limitation for this study is that there were no specific diagnostic criteria for a concussion for the acutely concussed group. Because the physicians diagnosing were not members of the investigative team, we do not know the criteria used for each participant with a concussion. It is, therefore, possible that any of those defined as a concussed subject may not have had a concussion if different diagnostic criteria were applied. It is also possible that a control participant had a concussion and did not know. Although we believe that these scenarios are unlikely, this would have produced a misclassification and could influence the results, most likely in favor of a null finding.

Additional studies need to be completed to explore the normative range of performance on this novel HMD test for a generally neurologically healthy population. Exploration into whether normative differences in performance exist by specific group (athlete type, gender, age, etc.) also need to be explored. Additional analyses using machine learning may also elucidate features that distinguish concussed from nonconcussed individuals or even those with various neurological impairments or diagnoses.

## Conclusion

Our results demonstrate that VR can be used to deliver a novel clinical peripheral vision test and produce corroborating findings with previously published studies on this topic. Our results, however, further elucidate specific deficits in peripheral vision processing after concussion. This is particularly important when designing rehabilitative programs to target specific deficits. VR offers a portable solution, a controlled environment, strong repeatability, sensor-based metrics of performance, and potential to be integrated within a telehealth model of care. These features are particularly important in military and rural environments, where specialist care is not typically available.

## Abbreviations Used

HMD: head-mounted device
VR: virtual reality
